# The Whole Mitochondrial Genome Sequence of *Dendrobium loddigesii* Rolfe, an Endangered Orchid Species in China, Reveals a Complex Multi-Chromosome Structure

**DOI:** 10.3390/genes15070834

**Published:** 2024-06-25

**Authors:** Wenjun Tong, Dandan Yu, Xiaojing Zhu, Zhifang Le, Hui Chen, Feilong Hu, Shengmin Wu

**Affiliations:** Nanjing Institute of Environmental Sciences, Ministry of Ecology and Environment, Nanjing 210042, China; twj@nies.org (W.T.); dan.d.yu@hotmail.com (D.Y.); zhuxiaojing@nies.org (X.Z.); lezhifang00@163.com (Z.L.); beisu423@yeah.net (H.C.); hfl@nies.org (F.H.)

**Keywords:** mitogenome, *Dendrobium loddigesii*, RNA editing, synteny, phylogeny

## Abstract

*Dendrobium loddigesii* is a precious traditional Chinese medicine with high medicinal and ornamental value. However, the characterization of its mitochondrial genome is still pending. Here, we assembled the complete mitochondrial genome of *D. loddigesii* and discovered that its genome possessed a complex multi-chromosome structure. The mitogenome of *D. loddigesii* consisted of 17 circular subgenomes, ranging in size from 16,323 bp to 56,781 bp. The total length of the mitogenome was 513,356 bp, with a GC content of 43.41%. The mitogenome contained 70 genes, comprising 36 protein-coding genes (PCGs), 31 tRNA genes, and 3 rRNA genes. Furthermore, we detected 403 repeat sequences as well as identified 482 RNA-editing sites and 8154 codons across all PCGs. Following the sequence similarity analysis, 27 fragments exhibiting homology to both the mitogenome and chloroplast genome were discovered, accounting for 9.86% mitogenome of *D. loddigesii*. Synteny analysis revealed numerous sequence rearrangements in *D. loddigesii* and the mitogenomes of related species. Phylogenetic analysis strongly supported that *D. loddigesii* and *D. Amplum* formed a single clade with 100% bootstrap support. The outcomes will significantly augment the orchid mitochondrial genome database, offering profound insights into *Dendrobium’*s intricate mitochondrial genome architecture.

## 1. Introduction

The genus *Dendrobium*, belonging to the *Orchidaceae* family, comprises approximately 1500 species worldwide, with about 80 species native to China [1]. The stems of several plants within this genus have been utilized for centuries in traditional Chinese medicine [2]. *D. loddigesii* is a perennial epiphytic herb in the *Orchidaceae* family. It is primarily distributed in provinces such as Guizhou, Guangxi, Yunnan, and Guangdong in China [3]. The stems of *D. loddigesii* are a commonly used and precious Chinese medicinal herb, which has anti-inflammatory, antimicrobial, antioxidant, antitumor, and immunomodulatory effects [3,4,5]. It mainly contains polysaccharides, alkaloids, and other chemical components [6]. Due to the low germination rate of *D. loddigesii* seeds in natural environments, as well as excessive harvesting and utilization, the germplasm resources and wild populations of *D. loddigesii* have rapidly decreased [7]. Therefore, it is necessary to analyze the genetic relationship through mitochondrial sequencing to provide a theoretical basis for the sustainable utilization and protection of *D. loddigesii* germplasm resources.

The plant mitochondrial genome exhibits semi-autonomous genetic characteristics and serves as a crucial cellular organelle for respiratory metabolism and the supply of energy for life activities [8,9]. Compared with other organelle genomes, the mitochondrial genome is relatively large and has frequent structural changes, such as genome rearrangement, repeat sequence recombination, transfer, deletion, and repetition of foreign DNA [10]. This complexity makes it more challenging to assemble the mitochondrial genome [11]. While most plant mitogenomes are typically circular, the actual physical architecture of these genomes shows a diverse array of structures, including circles, linear molecules, and intricate branching patterns [12,13]. Furthermore, investigations into the structure of plant mitogenomes are predominantly focused on model species, including *Arabidopsis thaliana* [14], tobacco (Y Sugiyama, 2005) [15], and maize [16], which limits the application of generalized inferences about the plant mitochondrial genome evolution and function. Currently, studies have found that *Dendrobium* mitochondria exhibit a multiple chromosome phenomenon. However, only four mitochondrial genomes of *Dendrobium* have been published, including *D. wilsonii*, *D. Henanense*, *D. wilsonii*, and *D. Henanense* [17]. Hence, more mitogenomes should be explored to enhance our understanding of mitochondrial structures of *Dendrobium*. In this study, we aimed to sequence, assemble, and annotate the complete mitogenome of *D. loddigesii*. A comprehensive analysis of repeat sequences, synonymous codon usage, and RNA editing was performed, and synteny and phylogenetic relationships were compared with several previously reported mitochondria genomes. Our findings aim to demonstrate that the plant mitochondrial genome comprises various sequence elements characterized by complex structures.

## 2. Materials and Methods

### 2.1. Plant Materials and DNA Sequencing

Fresh leaves of *D. loddigesii* were rapidly collected and preserved at −80 °C. Genomic DNA was extracted using the modified CTAB method [18]. The qualified DNA samples were then sequenced on the Illumina NovaSeq 6000 platform (Genepioneer Biotechnologies Co., Ltd.; Nanjing, China).

### 2.2. Assembly and Annotation

For acquiring a high-quality *D. loddigesii* mitochondrial genome, the second-generation data were used to extract high-quality reads, while the original third-generational data were employed for correction. Then, the genome assembly was conducted utilizing NextPolish v1.3.1 (https://github.com/Nextomics/NextPolish, accessed on 18 August 2023). The annotation process for the draft mitochondrial genome of *D. loddigesii* followed established procedures [19]. Encoded proteins and rRNAs were annotated through Blastn searches of published plant mitochondrial sequences at the National Center for Biotechnology Information (NCBI). Transfer RNA genes (tRNAs) were annotated using tRNA scan-SE [20]. An ORF finder was utilized to analyze open reading frames (ORFs), and an Organellar Genome DRAW was employed for the construction of the mitochondrial genome [21].

### 2.3. Repeat Sequences Analysis

The examination of repeat structures, including forward (F), reverse (R), complement (C), and palindromic (P) repeats, was carried out utilizing vmatch v2.3.0 software (http://www.vmatch.de/, accessed on 18 August 2023). The MicroSAtellite identification tool (Misa) was used to explore mitochondrial SSR [22]. The parameters employed for the analysis were as follows: mononucleotides repeated at least 8 times, dinucleotides repeated at least 5 times, trinucleotides repeated at least 4 times, and tetra-, penta-, and hexanucleotides repeated at least 3 times. Tandem repeats were found using Tandem Repeats Finder (http://tandem.bu.edu/trf/trf.submit.options.html, accessed on 18 August 2023) [23].

### 2.4. Synonymous Codon Usage Analysis

To assess the synonymous codon usage patterns within the mitochondrial genome, we employed the relative synonymous codon usage (RSCU) using the CodonW1.4.4 (http://codonw.sourceforge.net/, accessed on 16 August 2023) [24]. Subsequently, the R package (3.5.1) ggplot2 was utilized to generate visualizations of the RSCU data, providing a clear and informative representation of the codon usage patterns.

### 2.5. RNA Editing Analyses and Chloroplast to Mitochondrion DNA Transfer

To identify RNA-editing sites within the mitochondrial genes of *D. loddigesii*, the plant mitochondrial gene-encoding proteins were used as the reference proteins. The plant predictive RNA editor (PREP) suite (http://prep.unl.edu/, accessed on 16 August 2023) was employed to analyze the editing sites [25]. The chloroplast genome sequence of *D. loddigesii* (accession number: NC_036355.1) was retrieved from the NCBI Organelle Genome Resources Database. The homologous fragments were identified utilizing BLAST v2.10.1 software.

### 2.6. Synteny and Phylogenetic Analysis

A dot plot comparing pairwise sequences was generated to visualize conservative co-linear blocks. Furthermore, a multiple synteny plot was created to depict the mitogenome of *D. loddigesii* in comparison with related species. For phylogenetic tree analysis, we utilized the conserved PCGs extracted from the mitochondrial genome of *D. loddigesii* along with those from 15 other taxa. The 15 mitochondrial genomes were obtained from NCBI Organelle Genome Resources database, and the conserved PCGs were extracted using the Tbtools2.07 software. The gene sequences were then aligned using the Muscle v5 software, and a Neighbor-joining (NJ) tree was constructed using the Mega 11.0 software [26].

## 3. Results

### 3.1. Genomic Features of the D. loddigesii Mitogenome

**Figure 1:**
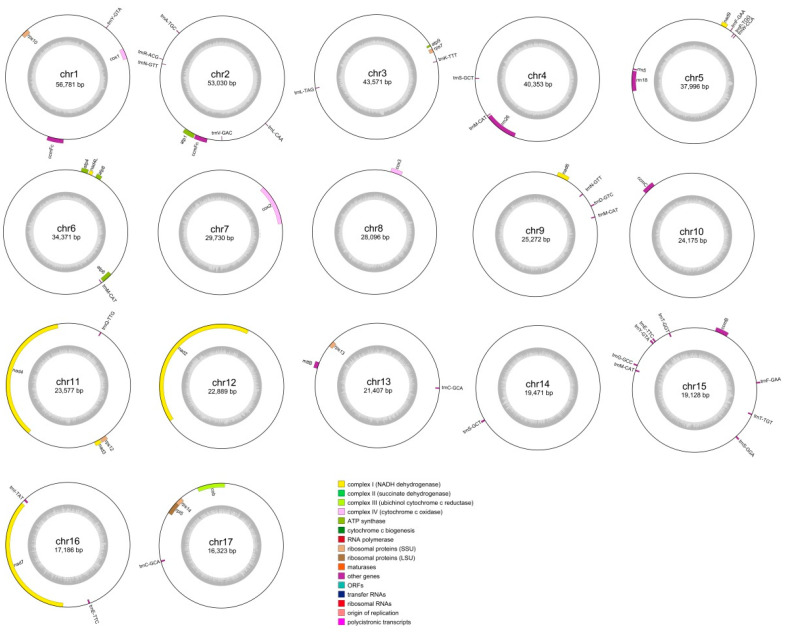
The *D. loddigesii* mitogenome map consists of 17 circular chromosomes.

We used Illumina and Nanopore sequencing platforms to acquire basic data to assemble the mitochondrial genome. Among them, raw Illumina data were 15.61 Gb and Nanopore raw data were 22.82 Gb, with an N50 of 21,378 bp and an average read length of 7140 bp (Supplementary Tables S1 and S2). By aligning the Illumina and Nanopore sequencing, the graphical assembly became 17 circular contigs (Figure 1, Table 1), which depicted the entire mitogenomes of *D. loddigesii*. These obtained contigs were referred to as chromosomes in this context. The total length of the *D. loddigesii* mitogenome was 513,356 bp, with a GC content of 43.41%. The two largest chromosomes, measuring 56,781 bp and 53,030 bp, respectively, comprised approximately 21.49% of the total size of the mitogenome. On the other hand, the smallest fragment was 16,323 bp and accounted for approximately 3.18% of the mitogenome. The mitochondrial genome had an average GC content of 43.46%, with the highest content reaching 45.93% and the lowest content being 38.27%.

The complete mitochondrial genome of *D. loddigesii* was annotated with 70 genes, including 36 PCGs, 31 tRNA genes, and 3 rRNA genes (Table S3). The PCGs can be classified as ATP synthases (*atp1*, *atp4*, *atp6*, *atp8*, and *atp9*), cytochrome c biogenesis (*ccmB*, *ccmC*, *ccmFc*, and *ccmFn*), ubiquinol cytochrome c reductases (1 gene, *cob*), maturases (*matR*), cytochrome C oxidases (*cox1*, *cox2*, and *cox3*), transport membrane proteins (*mttB*), NADH dehydrogenases (*nad1*, *nad2*, *nad3*, *nad4*, *nad4L*, *nad5*, *nad6*, *nad7*, and *nad9*), a large subunit of ribosome (*rpl5*), small subunits of ribosome (*rps10*, *rps12*, *rps13*, *rps14*, and *rps7*), and succinate dehydrogenase (*sdh4*) (Table 2). Among the 36 PCGs, 8 contained introns: *ccmFc* and *rps10* had a single intron, *cox2* contained two introns, *nad4* contained three introns, and *nad1*, *nad2*, *nad5*, and *nad7* had four introns. Among the 31 tRNA proteins, 7 tRNA genes (*trnC-GCA*, *trnE-TTC*, *trnL-GAA*, *trnM-CAT*, *trnN-GTT*, *trnS-GCT*, and *trnY-GTA*) showed the multi-copy phenomenon, of which 1 gene, *trnM-CAT* included up to 4 copies. The two genes, *trnA-TGC* and *trnI-TAT,* had an intron.

### 3.2. Repeat Sequence Analysis

**Figure 2:**
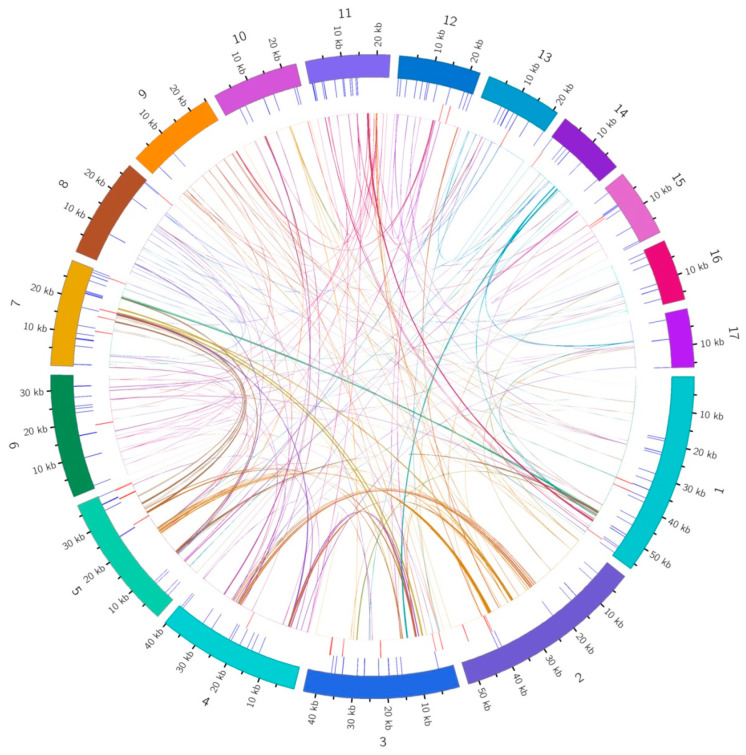
Repeated sequence distribution in the *D. loddigesii* mitogenome. The outermost circle was the SSRs, followed by tandem repeat sequences, and the innermost was the dispersed repeat sequences.

A total of 403 repeats were found in the *D. loddigesii* mitochondrial genome, including 221 dispersed sequences, 146 SSRs, and 36 tandem sequences (Figure 2, Table S4). The dispersed sequences included 90 forward repeat sequences and 131 palindromic repeat sequences (Table S5). The largest forward repeat sequence was 631 bp in length, while the palindromic repeat sequence had a size of 467 bp. The total length of the dispersed repeat sequences was 24,272 bp, accounting for 4.73% of the total length of the mitochondrial genome. Moreover, among 146 SSRs, 48 mononucleotide, 34 dinucleotide, 18 trinucleotide, 35 tetranucleotide, 8 pentanucleotide, and 3 hexanucleotide repeat types were explored. Among the mononucleotide repeat types, the repeats of A/T were the most ordinary, and only one type was C/C repeats (Table S6). A total of 36 tandem repeats were detected, ranging from 6 to 34 bp in length, with a matching degree surpassing 78% (Table S7). Chromosomes 3, 5, and 7 exhibited the highest occurrence of tandem repeats, whereas chromosomes 9, 10, 11, 16, and 17 displayed an absence of such repeats. This observation indicated an uneven distribution of tandem repeats across the mitochondrial genome of *D. loddigesii.*

### 3.3. Codon Usage Analysis

**Figure 3:**
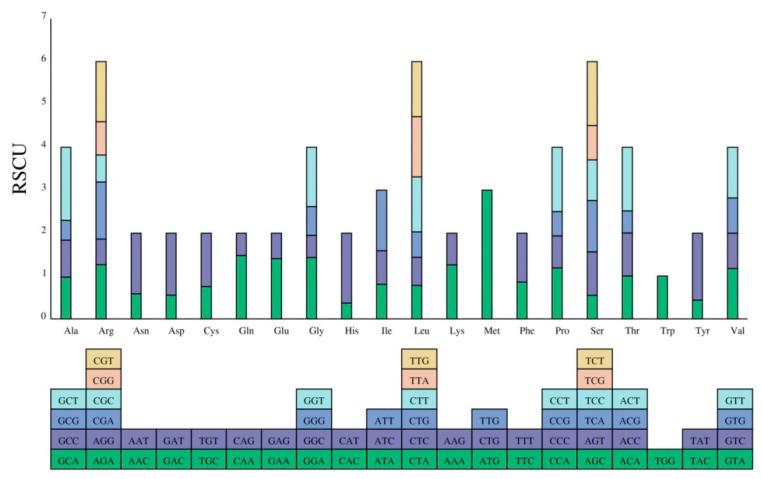
RSCU in the *D. loddigesii* mitogenome. The *x*-axis represents the different kinds of amino acids. The *y*-axis represents the value of RSCU.

The relative synonymous codon usage (RSCU) is generally thought to reflect the result of biological natural selection, with RSCU values exceeding 1 indicating a preference for specific amino acid codons [27]. Codon usage analysis was conducted on 36 PCGs within *D. lodigesii* mitochondria. The results revealed that all genes were encoded by 8154 codons, encoding 20 amino acids (Figure 3, Table S8). Notably, the RSCU values of 4338 codons were greater than 1, indicating that they were used more frequently. Furthermore, in addition to the high frequency of use of the three-stop codons, UAU, UGA, and UAG, a general preference for specific codons was observed in mitochondrial PCGs. For example, GCA, GCC, GCG, and GCU were the most commonly used codons in *D. lodigesii*.

### 3.4. RNA Editing Site Analysis

**Figure 4:**
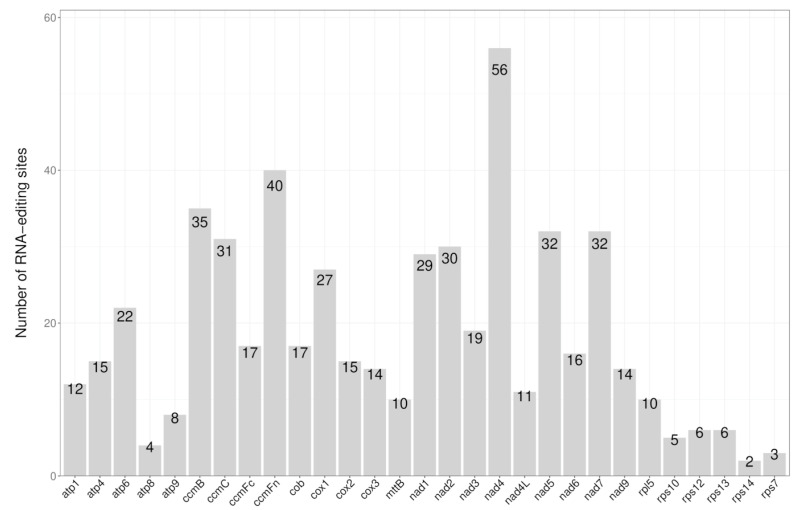
Distribution of RNA-editing sites in PCGs of the *D. loddigesii* mitogenome.

In higher plants, RNA editing is a post-transcriptional process necessary for mitochondrial gene expression [28]. In the *D. loddigesii* mitochondria, 538 RNA-editing sites were predicted in PCG genes (Figure 4). Among those PCG genes, the most RNA-editing sites were the nad4 gene (56 sites, 10.41%) followed by the ccmFn gene with 40 RNA editing sites. The minimum number of RNA-editing sites were the rps14 and rps7 genes, with only two and three editing sites, respectively. In addition, further analysis showed that 262 RNA-editing sites (48.70%) of the amino acids changed from hydrophilic to hydrophobic, 156 sites (29.00%) from hydrophobic to hydrophobic, 70 sites (13.01%) from hydrophilic to hydrophilic, 48 sites (8.92%) from hydrophobic to hydrophilic, and only 2 editing sites (0.37%) of amino acids became stop codons (X) (Table 3). The studies also found that 230 editing sides (42.74%) of the amino acids were changed to leucine (L), showing a leucine tendency.

### 3.5. Chloroplast to Mitochondrion DNA Transfer

**Figure 5:**
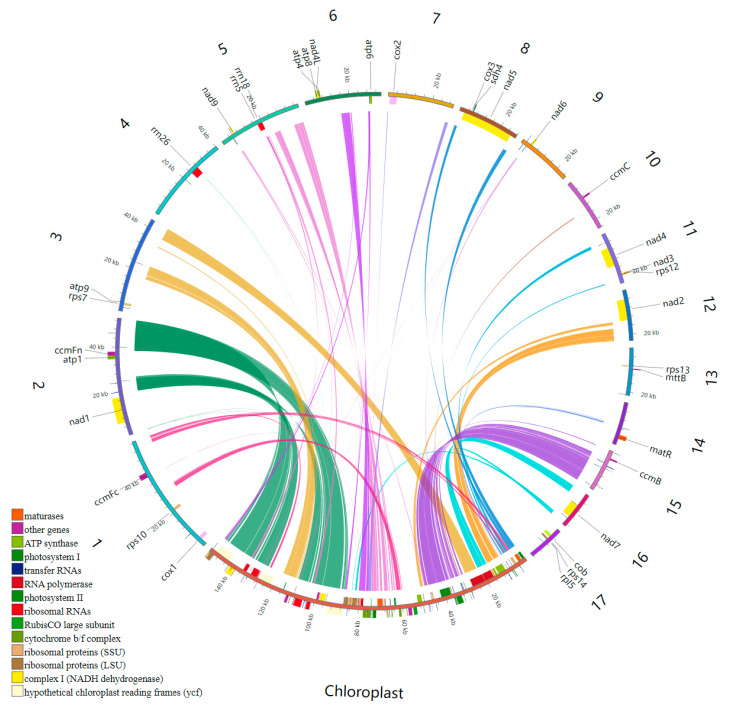
Homologous fragments were distributed between mitochondria and chloroplast in *D. loddigesii*.

Sequence similarity analysis showed that there were 27 homologous fragments shared between mitochondrial and chloroplast genomes, with a total length of 50,632 bp, accounting for 9.86% of the total length of the *D. lodigesii* mitogenome (Figure 5, Table 4). Among these, six fragments exceeded 1000 bp, with fragments 1 and 2 being the longest at 8595 bp, while the smallest fragment was 23 for 29 bp. Through annotation of these homologous sequences, 18 integrated chloroplast-derived genes were identified, specifically including *trnL-CAA*, *trnR-ACG*, *trnN-GUU*, *trnV-GAC*, *trnA-UGC*, *trnL-UAG*, *trnS-GGA*, *trnT-UGU*, *trnG-GCC*, *trnM-CAU*, *trnT-GGU*, *trnE-UUC*, *trnY-GUA*, *trnW-CCA*, *trnP-UGG*, *trnF-GAA*, *trnQ-UUG*, and *trnS-GCU*, and two incomplete rRNA genes (*rrn18* and *rrn26*) were also discovered. Notably, all transferred genes were tRNA genes and partial rRNA genes, and no PCGs were found, which indicated that tRNA genes were more conserved in the *D. lodigesii* plastid genome.

### 3.6. Synteny and Phylogenetic Analysis

**Figure 6:**
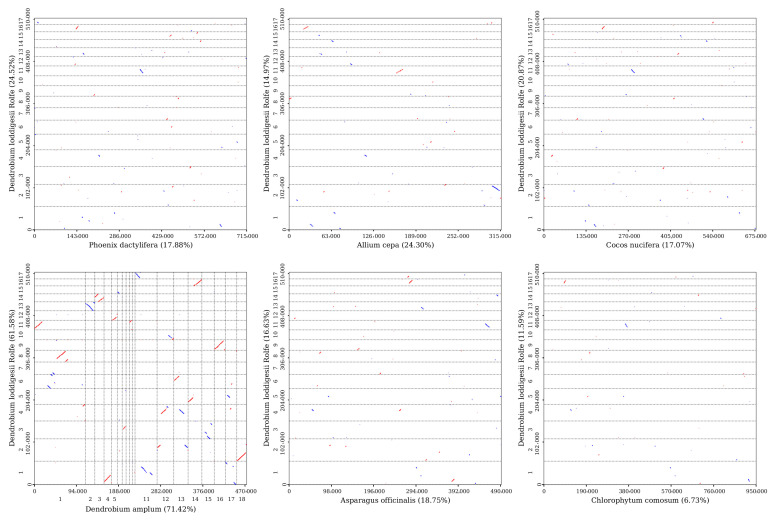
Dot plot graphs show similar sequences between mitogenomes primarily in *D. loddigesii* and related species. The red line in the box is a forward comparison, while the blue line is a reverse complementary comparison.

**Figure 7:**
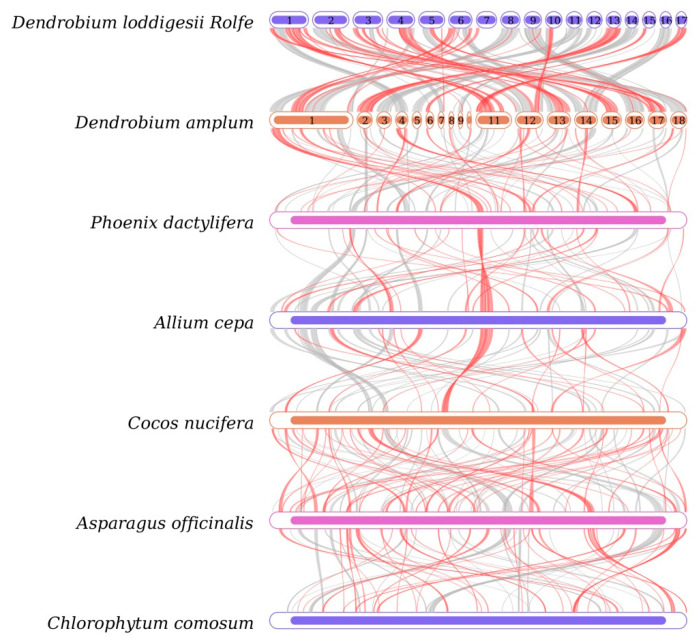
Collinearity plots of the mitogenomes of *D. loddigesii* and related species. The boxes in each row represent the mitogenomes, and the lines in the middle represent homologous regions.

Sequence similarity was used to map the multiple synteny plot of *D. lodigesii* with six related species (Table S9). A large number of syntenic collinear blocks were found between *D. lodigesii* and *D. amplum*. The dot plot analysis suggested that only sporadic collinear regions existed between the mitogenomes of *D. lodigesii* and the other five mitochondrial genomes (*Phoenix dactylifera*, *Allium cepa*, *Cocos nucifera*, *Asparagus officinalis*, and *A. officinalis*), showing poor collinearity (Figure 6). Moreover, the analysis also found that the co-linear blocks were not aligned identically in the mitogenomes of *D. lodigesii* and *D. Amplum* (Figure 7), which may suggest that the mitogenome of *D. lodigesii* had undergone substantial genomic rearrangements, resulting in a highly variable and unconserved structure.

**Figure 8:**
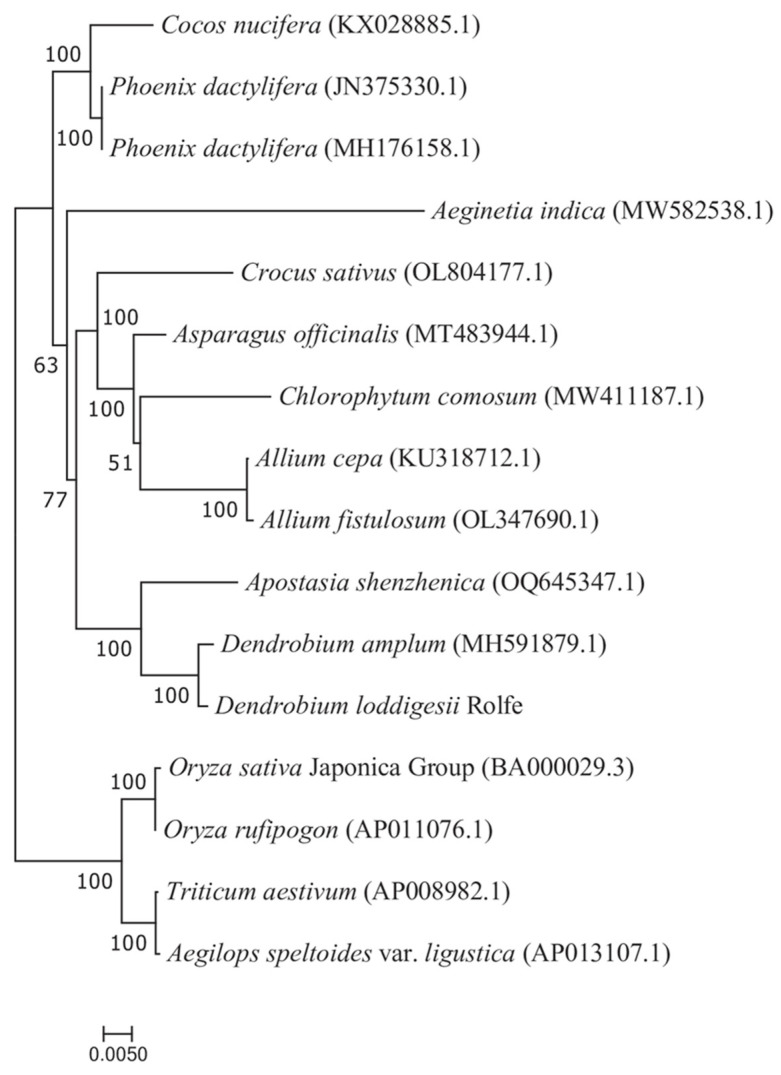
The phylogenetic relationships of *D. loddigesii* and other 15 species based on conserved mitochondrial genes.

To determine the phylogenetic location of *D. lodigesii*, the mitochondrial genome sequences of 15 angiosperms were retrieved from GenBank (Table S10). The sequences were based on 25 conserved PCGs, which were to establish a phylogenetic tree, using *Aegilops speltoides* as the outgroup (Figure 8). The phylogenetic tree provided robust evidence, with 100% bootstrap support, for a close phylogenetic affinity between *D. lodigesii* and *D. amplum*. Both species are native to south-central and south-eastern China, and phylogenetic analysis further confirms this geographical proximity.

## 4. Discussion

### 4.1. Characterization of the D. lodigesii Mitogenome

Mitochondria play an important role as organelles within eukaryotic cells [29]. Unlike animal cells, plant mitochondrial DNA exhibits amazing structural diversity, with its structure being able to rapidly switch between linear, circular, and branched forms within the organism [30]. Generally, plant mitochondria are usually assembled into a large circle structure, but their true morphology may consist of smaller circles combined with branching DNA molecules [31]. In this study, we assembled the mitochondria of *D. lodigesii*, which contained 17 chromosomes and exhibited a multi-chromosome structure. The *Dendrobium* genus is generally polychromosome, with the mitochondrial genome sequences of *D. wilsonii* and *D. henenense* possessing 22 and 24 independent chromosome structures, respectively [32]. The rapid acquisition or loss of chromosomes has been postulated as a pivotal evolutionary process that explains these observed disparities [16]. The *D. loddigesii* mitogenome was annotated with 70 genes, which was similar to the number in *D. wilsonii* (77) and *D. henanense* (83) [32]. However, the most significant differences among the three mitogenomes were primarily observed in the tRNA genes. Specifically, the mitogenome of *D. loddigesii* contained 31 tRNA genes, while the mitogenomes of *D. Wilsonii* and *D. henanense* had 33 and 40 tRNA genes, respectively. These findings indicated that *Dendrobium* species exhibited the greatest variation in the number of tRNA genes within their mitochondrial genomes.

### 4.2. The Repeat Sequences in the D. lodigesii Mitogenome

The repeat sequences are potentially important markers for population and evolutionary studies [33]. In this study, a total of 403 repeat sequences were identified in the *D. loddigesii* mitochondrial genome, including 146 SSRs. SSRs have the advantages of codominance, high reproducibility, and the requirement of a small amount of DNA template [34,35]. These features enable their application in various scenarios, including DNA fingerprinting, gene mapping, and marker-assisted breeding [36,37,38].

### 4.3. RNA Editing in the D. lodigesii Mitogenome

In plants, RNA editing is a pivotal process that significantly contributes to mitochondrial gene expression and functionality [39]. Numerous RNA editing events have the potential to introduce changes in RNA sequences, ultimately resulting in variations in the amino acid sequences of the translated protein products [40,41]. A total of 538 RNA-editing sites were detected in *D. loddigesii* mitochondria. Consistent with observations in other plant species [42,43,44], a majority of RNA-editing sites in this mitochondrion occurred at the first or second positions within the RNA sequence. RNA editing events at two specific sites lead to the creation of stop codons in *D. loddigesii* mitochondria, which is frequently linked to the production of proteins that exhibit high conservation to those identified in other species [40]. This mechanism facilitates efficient gene expression within mitochondria.

### 4.4. MTPTs in the D. lodigesii Mitogenome

Horizontal gene transfer (HGT) occurs between the genomes of organelles (such as plastids and mitochondria) and the nuclear genomes of plant cells, and it is a general phenomenon that significantly impacts plant evolution [45]. One of the more intriguing phenomena in plant genetics is the transformation of DNA fragments from plastids into mitochondrial genomes, referred to as plastid-to-mitochondrial transfers (MTPTs) [46]. These transfers involve the movement of genetic material, typically short DNA sequences, from the plastid genome into the mitochondrial genome [47]. In this study, eighteen complete tRNA genes, including *trnL-CAA, trnR-ACG*, *trnN-GUU*, *trnV-GAC*, *trnA-UGC*, *trnL-UAG*, *trnS-GGA*, *trnT-UGU*, *trnG-GCC*, *trnM-CAU*, *trnT-GGU*, *trnE-UUC*, *trnY-GUA*, *trnW-CCA*, *trnP-UGG*, *trnF-GAA*, *trnQ-UUG*, and *trnS-GCU*, were found to migrate from the chloroplast to the mitogenome in *D. lodigesii*. Interestingly, the mitogenome of *D. lodigesii* harbored only 13 native tRNA genes, suggesting that over half of its tRNA genes had undergone HGT from the chloroplast. During the entire evolutionary process, the horizontal transfer of tRNA genes from the chloroplast to the mitochondrion in *D. lodigesii* had resulted in the acquisition of functionally conserved tRNAs, which were prevalent across the angiosperms [48]. Among the tRNA genes’ horizontally transferred events, *trnW-CCA* frequently appeared in the mitochondrial genomes of diverse angiosperms [49,50]. Prior research had demonstrated that *trnM-CAU* possessed a potential functional role in plant mitochondria genomes, suggesting that it underwent transfer during an initial phase of evolutionary development [51]. Both tRNA genes, *trnW-CCA*, and *trnM-CAU*, were also discovered as part of the level gene transfer involving the organelles of *D. lodigesii*.

### 4.5. Synteny and Phylogenetic Analyses in the D. lodigesii Mitogenome

We conducted homologous collinear alignments to delve into the rearrangement and conservative sequence patterns within the mitochondrial genome. The findings indicated that *D. lodigesii* and several other genera (*Phoenix, Allium*, *Cocos*, *Asparagus*, and *Chlorophytum*) displayed low collinearity, whereas *D. amplum* exhibited high collinearity (71.42%). This suggests that closely related species tended to have longer collinear regions, while distantly related genomes showed poorer collinearity [52]. Moreover, analysis of collinear alignments revealed inconsistent alignment of collinear blocks in the mitogenomes of *D. lodigesii* and *D. Amplum*, which may suggest that the mitogenome had undergone substantial genomic rearrangements, resulting in a highly variable and unconserved structure. This outcome aligned with earlier findings from mitochondrial collinearity analysis of *D. Wilsonii* and *D. Henanense*, demonstrating a substantial presence of mitochondrial rearrangements in both *Dendrobium* species [32].

Here, we built the phylogeny of *Dendrobium* using conserved mitochondrial PCG sequences from 15 angiosperm species retrieved from GenBank. Unlike chloroplast and nuclear genomes, mitogenomes are rarely used for phylogenetic analysis in higher plants, primarily due to their slow mutation frequency, high rate of genome recombination, and integration of exogenous DNA [53,54]. However, in this study, the *D. lodigesii* clade was sister to the *D. Amplum* clade with strong support (100%) in the present study, and the issue of weakly supported nodes in mitochondrial gene trees has been well addressed. These results suggest that PCG genes in plant mitochondrial genomes can be used for phylogenetic analysis.

## 5. Conclusions

In this study, we have successfully assembled and annotated the mitogenome of an orchid plant, *D. loddigesii*, revealing a complex multi-chromosome structure. The total length of *D. loddigesii* mitogenome was 513,356 bp, which consisted of 17 circular chromosomes. The genome was annotated with 70 genes, including 36 PCGs, 31 tRNA genes, and 3 rRNA genes. The repeat sequences, codon preference, and RNA-editing sites were also characterized. In addition, we also identified MTPTs and performed synteny and phylogenetic analyses to gain a deeper insight into the evolutionary trajectory of the mitogenome in *Dendrobium*. The results of this research further validate the intricate structure of mitogenomes in the orchid family.

## Figures and Tables

**Table 1:** The information of the *D. loddigesii* mitogenome.

ID	Accession Number	Length (bp)	GC (%)
Circular 1	PP829175	56,781	44.42
Circular 2	PP829176	53,030	43.36
Circular 3	PP829177	43,571	41.19
Circular 4	PP829178	40,353	44.24
Circular 5	PP829179	37,996	43.02
Circular 6	PP829180	34,371	42.63
Circular 7	PP829181	29,730	43.32
Circular 8	PP829182	28,096	43.44
Circular 9	PP829183	25,272	44.40
Circular 10	PP829184	24,175	43.87
Circular 11	PP829185	23,577	45.93
Circular 12	PP829186	22,889	40.28
Circular 13	PP829187	21,407	45.55
Circular 14	PP829188	19,471	45.01
Circular 15	PP829189	19,128	38.27
Circular 16	PP829190	17,186	44.82
Circular 17	PP829191	16,323	45.08
Total	-	513,356	43.41

**Table 2:** Gene composition in the *D. loddigesii* mitogenome.

Group of Genes	Name of Genes
ATP synthase	*atp1*, *atp4*, *atp6*, *atp8*, *atp9*
Cytochrome c biogenesis	*ccmB*, *ccmC*, *ccmFc* *, *ccmFn*
Ubiquinol cytochrome c reductase	*cob*
Cytochrome c oxidase	*cox1*, *cox2* **, *cox3*
Maturases	*matR*
Transport membrane protein	*mttB*
NADH dehydrogenase	*nad1* ****, *nad2* ****, *nad3*, *nad4* ***, *nad4L*, *nad5* ****, *nad6*, *nad7* ****, *nad9*
Large subunit of ribosome	*rpl5*
Small subunit of ribosome	*rps10* *, *rps12*, *rps13*, *rps14*, *rps7*
Succinate dehydrogenase	*sdh4*
Ribosomal RNAs	*rrn18*, *rrn26*, *rrn5*
Transfer RNAs	*trnA-TGC* *, *trnC-GCA*(2), *trnD-GTC*, *trnE-TTC*(2), *trnF-GAA*(2), *trnG-GCC*, *trnI-TAT* *, *trnK-TTT*, *trnL-CAA*, *trnL-TAG*, *trnM-CAT*(4), *trnN-GTT*(2), *trnP-TGG*, *trnQ-TTG*, *trnR-ACG*, *trnS-GCT*(2), *trnS-GGA*, *trnT-GGT*, *trnT-TGT*, *trnV-GAC*, *trnW-CCA*, *trnY-GTA*(2)

Notes: * one intron, ** two intron, *** three intron, **** four intron; Gene (2): Number of copies of multi-copy genes.

**Table 3:** Prediction of RNA-editing sites in the *D. loddigesii* mitogenome.

Type	RNA Editing	Number	Percentage
Hydrophilic–hydrophilic	CAC (H) => TAC (Y)	8	
	CAT (H) => TAT (Y)	18	
	CGC (R) => TGC (C)	12	
	CGT (R) => TGT (C)	32	
	Total	70	13.01%
Hydrophilic–hydrophobic	ACA (T) => ATA (I)	5	
	ACG (T) => ATG (M)	8	
	ACT (T) => ATT (I)	4	
	CGG (R) => TGG (W)	34	
	TCA (S) => TTA (L)	77	
	TCC (S) => TTC (F)	35	
	TCG (S) => TTG (L)	44	
	TCT (S) => TTT (F)	55	
	Total	262	48.70%
Hydrophilic–stop	CGA (R) => TGA (X)	2	
	Total	2	0.37%
Hydrophobic–hydrophilic	CCA (P) => TCA (S)	8	
	CCC (P) => TCC (S)	14	
	CCG (P) => TCG (S)	6	
	CCT (P) => TCT (S)	20	
	Total	48	8.92%
Hydrophobic–hydrophobic	CCA (P) => CTA (L)	46	
	CCC (P) => CTC (L)	8	
	CCC (P) => TTC (F)	6	
	CCG (P) => CTG (L)	27	
	CCT (P) => CTT (L)	28	
	CCT (P) => TTT (F)	14	
	CTC (L) => TTC (F)	6	
	CTT (L) => TTT (F)	12	
	GCA (A) => GTA (V)	1	
	GCC (A) => GTC (V)	1	
	GCG (A) => GTG (V)	4	
	GCT (A) => GTT (V)	3	
	Total	156	29.00%
	All	538	100%

**Table 4:** Fragments transferred from chloroplast to mitochondria in *D. loddigesii*.

Fragments	Alignment Length (bp)	Identity (%)	CP Start	CP End	Mt Start	Mt End	Genes
1	8595	99.162	89,287	97,869	51,672	43,104	*trnL-CAA*
2	8595	99.162	138,714	147,296	43,104	51,672	*trnL-CAA*
3	6080	99.266	125,537	131,590	25,997	19,973	*trnR-ACG*, *trnN-GUU*
4	5009	99.406	104,993	109,983	19,973	24,944	*trnR-ACG*, *trnN-GUU*
5	3855	97.588	98,032	101,870	43,117	39,293	*trnV-GAC*
6	3855	97.588	134,713	138,551	39,293	43,117	*trnV-GAC*
7	892	97.534	131,912	132,795	19,983	19,101	*trnA-UGC*
8	892	97.534	103,788	104,671	19,101	19,983	*trnA-UGC*
9	564	91.844	111,119	111,682	23,525	22,986	*trnL-UAG*
10	4124	91.844	42,520	46,619	14,032	18,149	*trnS-GGA*, *trnT-UGU*
11	1332	98.836	36,289	37,620	8109	9435	*trnG-GCC*, *trnM-CAU*
12	526	95.627	31,811	32,336	6418	5908	*trnT-GGU*
13	438	97.26	31,381	31,810	7143	6706	*trnE-UUC*, *trnY-GUA*
14	393	79.898	47,966	48,331	1	368	*trnF-GAA* (partical: 72.60%)
15	728	85.714	149,723	150,413	29,180	28,493	*trnM-CAU*
16	728	85.714	86,170	86,860	28,493	29,180	*trnM-CAU*
17	466	84.335	65,206	65,666	4931	5365	*trnW-CCA*, *trnP-UGG*
18	887	74.183	101,742	102,605	19,636	18,778	*rrn18* (partical: 43.25%)
19	887	74.183	133,978	134,841	18,778	19,636	*rrn18* (partical: 43.25%)
20	419	79.475	48,009	48,396	5401	5794	*trnF-GAA*
21	329	90.274	6532	6847	3650	3977	*trnQ-UUG*
22	649	82.897	7774	8386	11,710	11,096	*trnS-GCU*
23	29	88.235	45,071	45,099	11,438	11,466	*trnS-GCU* (partical: 32.95%)
24	83	96.386	126,909	126,991	2873	2791	*trnN-GUU*
25	83	96.386	109,592	109,674	2791	2873	*trnN-GUU*
26	97	85.567	105,841	105,937	27,256	27,160	*rrn26* (partical: 2.83%)
27	97	85.567	130,646	130,742	27,160	27,256	*rrn26* (partical: 2.83%)

## Data Availability

The sequencing data for Illumina and Nanopore platforms and the mitogenome sequences have been deposited in NCBI (https://www.ncbi.nlm.nih.gov/, accessed on 28 May 2024) with accession numbers PRJNA1113802, PRJNA1113802, and PP829175-PP829191.

## Supplementary Materials

The following supporting information can be downloaded at: https://www.mdpi.com/article/10.3390/genes15070834/s1, Table S1: Statistics of Illumina sequencing data. Table S2: Statistics of Nanopore sequencing data. Table S3: The annotation of the *D. loddigesii* mitogenome. Table S4: The repeat sequences in the *D. loddigesii* mitogenome. Table S5: the dispersed sequences in the *D. loddigesii* mitogenome. Table S6: SSRs in the *D. loddigesii* mitogenome. Table S7: The tandem sequences in the *D. loddigesii* mitogenome. Table S8. Codon usage analysis in the *D. loddigesii* mitogenome. Table S9: Species list used for synteny analysis. Table S10: Species list used for phylogenetic analysis.
